# Supplementary material for: The Impact of Color Cues on Word Segmentation by L2 Chinese Readers: Evidence from Eye Movements
Source: Behav Sci (Basel). 2025 Jul 3;15(7):904. doi: 10.3390/bs15070904 (PMC12292455; doi:10.3390/bs15070904)
Supplement: Supplementary file 1 [file behavsci-15-00904-s001.zip › behavsci-3653517-supplementary.pdf]

Table S1. Summary of word-level statistical effects for Target region

Group	Contrast	Statistic	Target			
			FFD	*GD*	*TRT*	*RI*
Group * Embedded word plausibility		*b*	0	-0.04	0.04	-0.02
		SE	0.01	0.02	0.02	0.08
		t/z	-0.19	-2.12*	2.06*	-0.26
L1 Readers	Embedded word plausibility	*b*	-0.01	0	-0.02	-0.08
		SE	0.01	0.01	0.01	0.05
		t/z	-0.84	-0.18	-2.12*	-1.46
L2 Readers	Embedded word plausibility	*b*	-0.01	-0.04	0.01	-0.1
		SE	0.01	0.01	0.01	0.07
		t/z	-0.92	-2.86*	0.98	-1.52
Group * Mono-color vs. Embedded word segmentation		*b*	0.01	0.02	0.07	0.01
		SE	0.01	0.02	0.02	0.1
		t/z	0.63	0.71	3.34*	0.1
L1 Readers	Mono-color vs Embedded word segmentation	*b*	0	-0.03	-0.08	-0.1
		SE	0.01	0.01	0.01	0.06
		t/z	-0.47	-2.17*	-5.86*	-1.59
L2 Readers	Mono-color vs Embedded word segmentation	*b*	0	-0.01	-0.01	-0.09
		SE	0.01	0.02	0.02	0.08
		t/z	0.42	-0.83	-0.39	-1.19
Group * Mono-color vs. Incremental word segmentation		*b*	0.02	0.03	0	-0.05
		SE	0.01	0.02	0.02	0.1
		t/z	1.33	1.26	-0.16	-0.46
L1 Readers	Mono-color vs. Incremental word segmentation	*b*	0.02	0.02	-0.01	-0.06
		SE	0.01	0.01	0.01	0.06
		t/z	1.75⁺	1.48	-1.02	-1.04
L2 Readers	Mono-color vs. Incremental word segmentation	*b*	0	-0.01	-0.01	-0.02
		SE	0.01	0.02	0.02	0.08
		t/z	-0.3	-0.44	-0.6	-0.29
Group * Incremental word segmentation vs.		*b*	-0.03	-0.04	-0.07	0.04
		SE	0.01	0.02	0.02	0.1
		t/z	-1.97*	-1.97*	-3.19*	0.37

Embedded word segmentation						
L1 Readers	Incremental word segmentation vs. Embedded word segmentation	*b*	-0.02	-0.05	-0.07	-0.03
		SE	0.01	0.01	0.01	0.06
		t/z	-2.25*	-3.67*	-4.87*	-0.55
L2 Readers	Incremental word segmentation vs. Embedded word segmentation	*b*	0.01	-0.01	0	-0.07
		SE	0.01	0.02	0.02	0.08
		t/z	0.73	-0.4	0.21	-0.9
Group * Plausibility * Mono-color vs. Embedded word segmentation		*b*	0	-0.06	-0.11	-0.38
		SE	0.03	0.04	0.04	0.2
		t/z	-0.15	-1.5	-2.57*	-1.92+
Group * Plausibility * Mono-color vs. Incremental word segmentation		*b*	-0.01	-0.05	0.03	0.34
		SE	0.03	0.04	0.04	0.2
		t/z	-0.4	-1.12	0.74	1.71+
Group * Plausibility * Incremental word segmentation vs. Embedded word segmentation		*b*	0.02	0.11	0.08	0.04
		SE	0.03	0.04	0.04	0.19
		t/z	0.56	2.63*	1.83+	0.21

Converged model for FFD ,epvar.lmer = lmer(depvar ~ PI * COLOR * GROUP + (1 + PI|participant) + (1|item), datafile)
Converged model for GD, TRT: depvar.lmer = lmer(depvar ~ PI * COLOR*GROUP + (1|participant) + (1|item), datafile)
Converged model for RI: depvar.glmer = glmer(depvar ~ PI * COLOR*GROUP + (1|participant) + (1|item), datafile, family = binomial)

Table S2. Summary of word-level statistical effects for Pre-target region

Group	Contrast	Statistic	Pre-target			
			FFD	*GD*	*TRT*	*RI*
Group * Embedded word plausibility		*b*	0.01	0.08	0.11	0.16
		SE	0.01	0.02	0.02	0.09
		t/z	0.82	5.04*	5.90*	1.86
L1 Readers	Embedded word plausibility	*b*	0.01	0.03	0.04	0.09
		SE	0.01	0.01	0.01	0.06
		t/z	1.83^{+}	2.88*	3.34*	1.66^{+}
L2 Readers	Embedded word plausibility	*b*	0.02	0.11	0.15	0.25
		SE	0.01	0.01	0.01	0.07
		t/z	2.68*	9.17*	10.66*	3.82*
Group * Mono-color vs. Embedded word segmentation		*b*	0.01	0.03	0.09	0.17
		SE	0.01	0.02	0.02	0.11
		t/z	0.37	1.55	3.81*	1.53
L1 Readers	Mono-color vs Embedded word segmentation	*b*	0.02	0	-0.02	-0.05
		SE	0.01	0.01	0.01	0.07
		t/z	1.69^{+}	0.32	-1.14	-0.76
L2 Readers	Mono-color vs Embedded word segmentation	*b*	0.02	0.03	0.07	0.12
		SE	0.01	0.01	0.02	0.08
		t/z	1.94^{+}	2.28*	3.99*	1.46
Group * Mono-color vs. Incremental word segmentation		*b*	-0.02	-0.01	-0.06	-0.14
		SE	0.01	0.02	0.02	0.11
		t/z	-1.31	-0.4	-2.82*	-1.31
L1 Readers	Mono-color vs. Incremental word segmentation	*b*	0.01	0.01	-0.01	-0.18
		SE	0.01	0.01	0.01	0.07
		t/z	0.83	0.84	-0.93	-2.64*
L2 Readers	Mono-color vs. Incremental word segmentation	*b*	0.03	0.02	0.05	-0.04
		SE	0.01	0.01	0.02	0.08
		t/z	2.45*	1.24	2.93*	-0.44
		b	0.01	-0.02	-0.02	-0.03

Group * Incremental word segmentation vs. Embedded word segmentation		*SE*	0.01	0.02	0.02	0.11
		t/z	0.94	-1.16	-0.99	-0.24
L1 Readers	Incremental word segmentation vs. Embedded word segmentation	*b*	0.01	-0.01	0	0.13
		SE	0.01	0.01	0.01	0.07
		t/z	0.87	-0.54	-0.22	1.91^{+}
L2 Readers	Incremental word segmentation vs. Embedded word segmentation	*b*	-0.01	0.02	0.02	0.15
		SE	0.01	0.01	0.02	0.08
		t/z	-0.52	1.04	1.06	1.90^{+}
Group * Plausibility * Mono-color vs. Embedded word segmentation		*b*	0.01	0.07	0.05	-0.37
		SE	0.03	0.04	0.05	0.22
		t/z	0.45	1.78^{+}	1.03	-1.74^{+}
Group * Plausibility * Mono-color vs. Incremental word segmentation		*b*	-0.01	-0.09	-0.01	0.47
		SE	0.03	0.04	0.05	0.21
		t/z	-0.17	-2.35*	-0.31	2.21*
Group * Plausibility * Incremental word segmentation vs. Embedded word segmentation		*b*	-0.01	0.02	-0.03	-0.1
		SE	0.03	0.04	0.05	0.21
		t/z	-0.28	0.57	-0.72	-0.45

Converged model for FFD ,epvar.lmer = lmer(depvar ~ PI * COLOR * GROUP + (1 + PI|participant) + (1|item), datafile)
Converged model for GD, TRT: depvar.lmer = lmer(depvar ~ PI * COLOR*GROUP + (1|participant) + (1|item), datafile)
Converged model for RI: depvar.glmer = glmer(depvar ~ PI * COLOR*GROUP + (1|participant) + (1|item), datafile, family = binomial)
